# Isoform-specific *NF1* mRNA levels correlate with disease severity in Neurofibromatosis type 1

**DOI:** 10.1186/s13023-019-1223-1

**Published:** 2019-11-15

**Authors:** Antonia Assunto, Ursula Ferrara, Alessandro De Luca, Claudia Pivonello, Lisa Lombardo, Annapina Piscitelli, Cristina Tortora, Valentina Pinna, Paola Daniele, Rosario Pivonello, Maria Giovanna Russo, Giuseppe Limongelli, Annamaria Colao, Marco Tartaglia, Pietro Strisciuglio, Daniela Melis

**Affiliations:** 1 Department of Translational Medical Science, Section of Pediatrics, Federico II University, Via Sergio Pansini, 5, 80131 Naples, Italy; 2 IRCCS Casa Sollievo della Sofferenza, Molecular Genetics Unit, San Giovanni Rotondo, Foggia, Italy; 3 Dipartimento di Medicina Clinica ed Endocrinologia, Università degli Studi di Napoli, “Federico II”, Naples, Italy; 4 Department of Molecular Medicine and Medical Biotechnology Federico II University, Naples, Italy; 5 Università della Campania “Luigi Vanvitelli”, AORN Colli, Ospedale Monaldi, Naples, Italy; 6 Genetics and Rare Diseases Research Division, Ospedale Pediatrico Bambino Gesù, Rome, Italy

**Keywords:** NF1, Neurofibromatosis type 1, Alternative splicing, Gene expression, mRNA isoforms, Phenotypic expressivity, Clinical variability

## Abstract

**Background:**

Neurofibromatosis type 1 (NF1) is characterized by an extreme clinical variability both within and between families that cannot be explained solely by the nature of the pathogenic *NF1* gene mutations. A proposed model hypothesizes that variation in the levels of protein isoforms generated via alternative transcript processing acts as modifier and contributes to phenotypic variability.

**Results:**

Here we used real-time quantitative PCR to investigate the levels of two major *NF1* mRNA isoforms encoding proteins differing in their ability to control RAS signaling (isoforms I and II) in the peripheral blood leukocytes of 138 clinically well-characterized NF1 patients and 138 aged-matched healthy controls. As expected, expression analysis showed that *NF1* isoforms I and II levels were significantly lower in patients than controls. Notably, these differences were more evident when patients were stratified according to the severity of phenotype. Moreover, a correlation was identified when comparing the levels of isoform I mRNA and the severity of NF1 features, with statistically significant lower levels associated with a severe phenotype (i.e., occurrence of learning disability/intellectual disability, optic gliomas and/or other neoplasias, and/or cerebrovascular disease) as well as in patients with cognitive impairment.

**Conclusions:**

The present findings provide preliminary evidence for a role of circuits controlling *NF1* transcript processing in modulating NF1 expressivity, and document an association between the levels of neurofibromin isoform I mRNA and the severity of phenotype and cognitive impairment in NF1.

## Background

Alternative splicing, the mechanism by which eukaryotic cells generate multiple RNAs from a single transcript, maximizes genome plasticity and versatility by promoting diversification of protein function and its spatiotemporal control [1–3]. In humans, as many as 92–94% of multiexon genes are predicted to undergo alternative splicing [4]. This process is important in the control of developmental programs and cell physiology, as well as in the pathogenesis and progression of human diseases [5]. It has been proposed that alternative splicing contributes to the clinical variability of Mendelian disorders by altering either the level of correctly spliced RNA pools or the ratio of different mRNA isoforms that result from transcript processing [6].

Neurofibromatosis 1 (NF1 [MIM: 162200]) is the most common non-chromosomal disorder affecting development and predisposing to cancer (approx. 1:2000–3000 live births) [7, 8]. It is transmitted as an autosomal dominant trait, and is caused by germline loss-of-function mutations in the *NF1* gene, which encodes neurofibromin, a GTPase negatively controlling RAS signaling [9]. Major features of NF1 include café-au-lait macules, skinfold freckling, and Lisch nodules of the iris, cutaneous and/or subcutaneous neurofibromas, variable learning disability/cognitive deficit (LD/CD), skeletal defects, and an increased risk for certain malignancies [10]. The neoplastic risk is related to functional loss of the GTPase activity of neurofibromin due to somatic hits involving *NF1*, according to the Knudson’s model, causing cell autonomous, and sustained activation of the Ras-mitogen-activated protein kinases (MAPK) pathway.

While NF1 is characterized by complete penetrance, variable expressivity is observed, with marked clinical variability even within families [11]. Phenotypic studies of large cohorts suggest that the type of mutation in the *NF1* gene generally does not correlate with the observed phenotypic variation [12, 13]. Exceptions are the constitutional *NF1* microdeletions [14] and missense variants affecting codons 844–848, which are associated with a more severe presentation [15], and the missense variants at codon 1809 [16, 17] and the 3-bp in-frame deletion, c.2970_2972del (p.Met992del), which conversely, are associated with a mild, mainly cutaneous, clinical presentation [18, 19]. Overall, the genotype-phenotype correlations identified so far have been reported to explain only a small amount of the extent of clinical variability characterizing the NF1 population [15, 19], and other factors, including stocastic events (e.g., second hits), and the genetic background (variation in modifier genes) are expected to contribute to a much larger fraction of the NF1 phenotipic variability [11]. The evidence that modifier genes contribute to the phenotypic expression of NF1 is strongly supported by familial studies [11, 13, 20, 21], which show that, independently by the *NF1* mutation, the grade of phenotypic concordance between members of the same family correlates with the genetic background, and that the relative contribution of the genetic background varies depending from the feature in question [11, 20]. Further evidence that genetic modifiers are major contributor to the variable expression of *NF1* comes from studies on animal models showing that *Nf1*^+/−^ mice strains have differences in phenotype severity with regards to the learning and behavioral aspects of the phenotype, as well as in the susceptibility to form astrocytomas [22–25]. Notably, by assessing *Nf1* mRNA levels in these models, it was also shown that trans-acting events modulate the phenotypic expression by impacting *Nf1* gene expression [26].

*NF1* is a large gene and its pre-mRNA undergoes alternative splicing. Several alternative exons that do not alter the reading frame of the gene have been identified, including 9a/9br, 10a-2, and 48a [6]. Of particular interest is exon 23a, which lies within the GAP-related domain (GRD) of neurofibromin, and is predominantly retained in most tissues**,** but specifically skipped in central nervous system neurons in humans [27, 28]. Of note, the two neurofibromin isoforms including/lacking the short amino acid stretch encoded by this exon differ in their ability to control Ras function [27, 28]. Isoform I, which lacks exon 23a, has ten times higher Ras-GAP activity than isoform II, in which exon 23a is retained. Biological importance of this exon during development has consistently been underlined by the observation that the mouse model in which exon 23a is constitutively deleted has a learning phenotype [24]. These considerations suggest an intriguing hypothesis in which changes in the levels of protein isoforms generated via alternative transcript processing, including alternative splicing, acts as genetic modifier in NF1 patients.

In the present study, we analyzed the levels of neurofibromin isoform I and II in circulating leukocytes of a cohort of genetically and clinically characterized NF1 patients stratified according to the severity of the phenotype, and correlated their expression levels with disease severity to assess whether alternative splicing may contribute to the variable expression characterizing NF1.

## Patients and methods

### Aim, design and setting of the study

One hundred and thirty eight individuals diagnosed with NF1 according to National Institutes of Health criteria were enrolled into the study at the Department of Translational Medicine, Federico II University of Naples, Pediatric Section, after the study protocol was discussed with each patient (or legal tutor) and an informed consent was signed. Patients’ clinical data were obtained from medical records over the past 20 years.

Collected clinical information included family history, and presence or absence of cafè-au-lait macules (CALMs), intertriginous skin freckling, Lisch nodules, cardiovascular malformations, skeletal malformations, endocrine system involvement, developmental delay (DD)/intellectual disability (ID), cerebrovascular malformations, cutaneous and subcutaneous neurofibromas (NFs), plexiform neurofibromas (PNFs), spinal neurofibromas, optic pathway gliomas (OPGs), and occurrence of other neoplasms (e.g., central nervous system gliomas, malignant peripheral nerve sheath tumors –MPNSTs-, juvenile myelomonocytic leukemia, rhabdomyosarcoma, phaeochromocytoma, gastrointestinal stromal tumours, juvenile xanthogranuloma, and lipoma). On the basis of clinical features, patients were divided into three groups according to the severity of the phenotype using the classification proposed by Riccardi [29]. The levels of *NF1* mRNA isoforms were investigated in peripheral blood leukocytes of patients and sex- and age-matched controls. All patients were screened for *NF1* and *SPRED1* mutations by parallel sequencing of the whole coding region and intronic stretches flanking splice sites (± 10 bp). Structural rearrangements were assessed by MLPA analysis using the MRC-Holland P295 probe set.

A comprehensive NF1 database with clinical and genetic data was built up. Genotype-phenotype correlations were investigated for each common clinical abnormality individually and for three groups of severity of disease.

### Study population

The study cohort included 17 families segregating the trait (12.31%) and 121 sporadic cases (87.69%) resulting from de novo mutations. Sixty-eight patients were males and 70 were females. The average age at time of diagnosis was 6.7 years (range 0.3–45 years), whereas the avarage age at observation was 16.4 years (range 0.60–55.90 years). Forty patients were children (aged between 0.6 and 11 years), 34 were in pubertal age (aged between 12 and 16), and 64 were adults (aged between 17 and 55.9).

Patients presenting with CALMs, axillary freckling, Lisch nodules, dermal and/or nodular neurofibromas, and non-progressive scoliosis were classified as “mild”, those presenting with plexiform neurofibromas, skeletal malformation, precocious or progressive scoliosis were classified as “moderate”, and patients with LD/CD, optic glioma and/or other neoplasms, and/or cerebrovascular disease were classified as “severe”. According to this stratification, 49 patients were classified as having a mild phenotype, 43 with as moderate phenotype and 46 as showing a severe phenotype. Demographic and clinical characteristics of the whole study cohort and subcohorts are reported in Table 1. A pathogenic or likely pathogenic *NF1* variant was found in 106/138 (76.8%) of the cases (see Additional file 1: Table S1 for details).

**Table 1:** Demografic and clinical characteristics of the 138 patients with NF1 included in the study

Feature	Mild phenotype (*n* = 49)	Moderate (*n* = 43)	Severe phenotype (*n* = 46)	Whole cohort (*n* = 138)
Mean age (average)	21.1 years (2.2–55.9 years)	14.6 years (1.2–36.4 years)	12.9 years* (0.6-53.5 years)	16.4 years (0.60–55.90 years)
No mutation	*N* = 13	*N* = 8	*N* = 10	*N* = 30
Gender	M = 19; F = 30	M = 27; F = 16	M = 22; F = 24	M = 68; F = 70
CALMs	49 (100%)	43 (100%)	46 (100%)	138
Lisch nodules	18 (36.8%)	20 (46,5%)	25 (54.4%)	63 (45,6%)
Axillary and/or inguinal freckling	37 (75.5%)	37 (86.0%)	33 (71.8%)	107 (77,5%)
Plexiform neurofibroma	0 (0.0%)	9 (20.9%)	9 (19/6%)	18 (13%)
Mild non-progressive scoliosis	21 (42.9%)	20 (46.5%)	NA	41 (29,7%)
Progressive scoliosis	0 (0.0%)	18 (41.8%)	25 (54.4%)	43 (31,1%)
Heart involvement	7 (14.3%)	9 (20.9%)	12 (26.1%)	28 (20,2%)
OPG	0 (0.0%)	0 (0.0%)	27 (58.7%)	27 (19,5%)
Other tumors	0 (0.0%)	0 (0.0%)	17 (37.0%)	17 (12,3%)
Development delay and/or cognitive deficit	0 (0.0%)	0	28 (60.9%)	28 (20,2%)

*for 45 living subjects; *F* females, *M* males, *NA* not available

### Expression studies

Relative expression of *NF1* isoforms I and II was assessed using TaqMan-based real-time quantitative PCR (RT-qPCR) assays, according to manufacturer’s recommendations (Thermo Fisher Scientific, Waltham, MA, USA). These assays were specific for *NF1* isoforms I and for isoform I + II, respectively. The expression value of isoform II was reported either as the sum of the expression value of both isoforms I and II pools and as isoform II data obtained subtracting the expression value of isoform I from the sum of the expression value of both isoforms I and II pools. Primers for RT-qPCR were purchased as assay-on demand (Thermo Fisher Scientific). Peripheral blood mononuclear cell samples obtained from patients and healthy controls were prepared from EDTA-anticoagulated blood by Ficoll-Hypaque density gradient centrifugation. Total RNA was extracted using TRIzol (Invitrogen Corporation, Carlsbad, CA, USA), according to the manufacturer’s instructions. Reverse transcription of first-strand cDNA was performed using oligo dT and the High-Capacity cDNA Archive Kit (Thermo Fisher Scientific), starting from 500 ng of RNA as a template. Beta-2-microglobulin (*B2M*) housekeeping gene was used as internal control. *NF1* and *B2M* mRNA pools were amplified from 100 ng of cDNA using the TaqMan Gene Expression PCR Master Mix (Thermo Fisher Scientific), according to the manufacturer’s instructions. Samples were run in duplicate, and mRNA levels were determined by comparing the expression of the two *NF1* isoforms with that of *B2M* internal control. Real-time qPCR was performed with an ABI 7900 Real-Time PCR instrument (Thermo Fisher Scientific). The data were analyzed with the SDS relative quantification software version 1.2.1 (Thermo Fisher Scientific). Relative quantification was performed using the Pfaffl method [30]. To ensure reliability of the data, 20 randomly selected patients were reanalyzed by RT-qPCR after one-year interval, proving high reproducibility of the data.

### Statistical analysis

Pearson’s correlation coefficients were used for the association studies. Group means (*NF1* isoforms I and II expression levels) were compared between groups by t-test for unpaired data. All statistical analyses were undertaken using the Statistical Package for the Social Sciences Software (SPSS) version 22 (IBM Corp., Armonk, NY, USA). A *p*-value less than 0.05 was considered significant. For statistical analysis comparing different groups of patients (namely patients with mild, moderate and severe phenotye), exclusively data from patients with diagnosis confirmed by molecular analysis were included.

## Results

Expression levels of *NF1* mRNA isoforms I and II were examined in peripheral blood leukocytes of 138 NF1 patients and compared with those of 138 population-, age- and sex-matched healthy controls. The expression value of isoform II was reported either as the sum of the expression value of both isoforms I and II pools and as isoform II data obtained subtracting the expression value of isoform I from the sum of the expression value of both isoforms I and II pools. Levels of *NF1* isoforms I, isoform (I + II) and isoform II are reported in Table 2.

**Table 2:** Comparative analysis of the expression levels of *NF1* isoforms I and II in peripheral blood leukocytes of NF1 patients and healthy controls stratified for the severity of the phenotype, and for the presence or absence of LD/MR. Mean value ±SE are reported

	NF1 patients	Healthy controls	*P* value
Total	138	138	
Isoform I	0.00066 ± 0.0001	0.0012 ± 0.0007	5.47E-06
Isoform II	0.0024 ± 0.001	0.01 ± 0.004	0.0004
Isoform II + I	0.0029 ± 0.0002	0.019 ± 0.003	0.0004
Isoform II/I	4.39 ± 1.9	4.05 ± 1.3	0.5
	Patients with severe phenotype	Patients with mild phenotype	
Total	36	36	
Isoform I	0.0004 ± 0.0001	0.0008 ± 0.0001	0.002
Isoform II	0.0017 ± 0.0018	0.0025 ± 0.001	0.09
Isoform II + I	0.0017 ± 0.001	0.0029 ± 0.0002	0.6
Isoform II/I	5.93 ± 1.7	3.92 ± 1.8	0.14
	Patients with LD/MR	Patients without LD/MR	
Total	26	45	
Isoform I	0.0004 ± 0.00009	0.0007 ± 0.0002	0.038
Isoform II	0.0015 ± 0.0005	0.0024 ± 0.0008	0.11
Isoform II + I	0.0019 ± 0.0003	0.0029 ± 0.0005	0.09
Isoform II/I	4.92 ± 1.7	4.2 ± 1.8	0.60
	Pediatric patients with LD/MR	Pediatric patients without LD/MR	
Total	22	31	
Isoform I	0.0001 ± 0.00008	0.0007 ± 0.0002	0.02
Isoform II	0.0014 ± 0.0004	0.0026 ± 0.001	0.10
Isoform II + I	0.0015 ± 0.0003	0.0029 ± 0.001	0.06
Isoform II/I	4 ± 1.4	3.72 ± 1.6	0.88

*LD/CD* learning disability/cognitive deficit

### Data analysis of isoform (I + II) provided results and statistical significance consistent with isoform II assessment

**Fig. 1:**
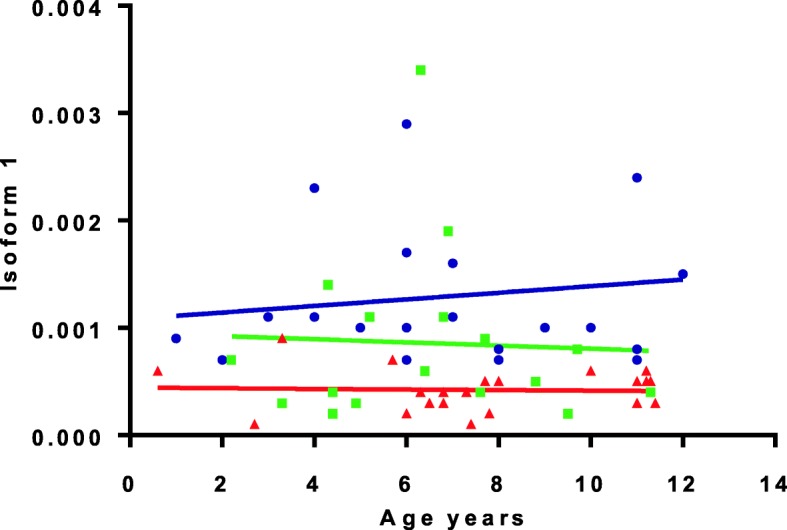
Comparison of *NF1* mRNA levels according to specific age between children with mild and (Green square) severe (Red triangle) phenotype and controls (Blue circle)

**Fig. 2:**
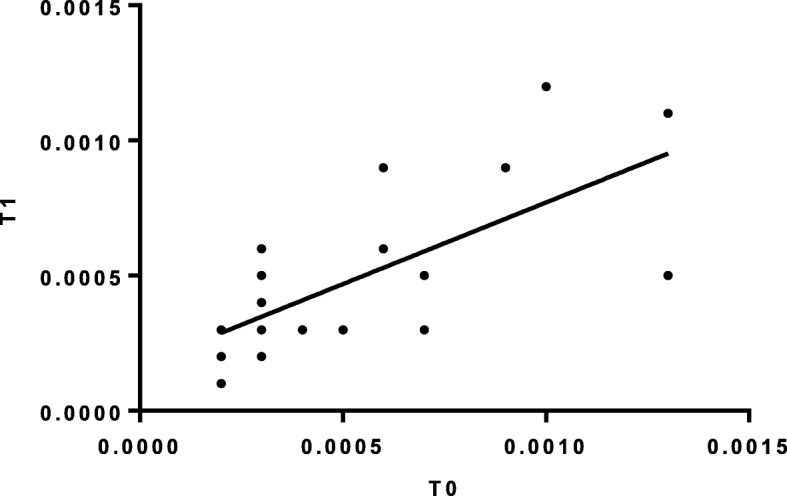
Results of the assays directed to analyze isoform 1 levels which were replicated in an unselected subgroup of patients randomly choose after 2 years

As expected, the analysis showed that the expression levels of both isoforms I and II were significantly lower in patients compared to controls (isoform I: *p* = 5.47E-06; isoform II: *p* = 0.0004). These differences remained significant when comparisons were made between healthy controls and patients subdivided according to disease severity (Table 2). Assessment of a possible correlation between the expression levels of *NF1* isoforms and the severity of disease documented a significant association between the expression level of isoform I and disease severity (linear association 6.2, *p* = 0.01). In particular, the expression level of isoform I was inversely correlated with disease severity either considering the entire cohort (Pearson r = − 0.247, *p* = 0.012), or when considering exclusively pediatric patients (Pearson r = − 0.427, *p* = 0.01). Subsequently, we specifically analyzed the expression level of *NF1* isoform I in severe cases respect to cases with moderate and mild phenotypes. Analysis confirmed that the expression level of isoform I was consistently reduced in the former. Similar results were obtained either considering the whole cohort (*p* = 0.002) or when only pediatric patients were included in the analysis (*p* = 0.002) (Fig. 1). Notably, the isoform II/isoform I ratio was higher in patients with severe phenotype althought it did not reach statistical significance (Table 2), suggesting a possible contribution of altered transcript processing to phenotypic expressivity. In order to evaluate the reproducibility of the data, the assays directed to analyze isoform 1 and isoform II levels were replicated in an unselected subgroup of patients randomly choosen after 2 years (Fig. 2).

To assess a possible differential contribution of specific clinical features defining the severe phenotype with the observed association, the levels of *NF1* mRNA isoforms were compared between groups taking into account LD/CD, neoplasias and cerebrovascular disease. Remarkably, patients with LD/CD showed significantly lower levels of isoform I than patients without LD/CD (*p* = 0.038). Importantly, this association remained significant after excluding from the analysis the affected subjects with *NF1* microdeletion (*p* = 0.039) or when only pediatric patients were considered (*p* = 0.02). This observation is relevant since by definition, patients with *NF1* microdeletion are haploinsufficient and display higher prevalence of LD/CD respect to the general NF1 population. By contrast, no significant association was found between *NF1* isoform expression and presence of neoplasias (*P* = 0.22) or cerebrovascular disease (*P* = 0.98).

**Fig. 3:**
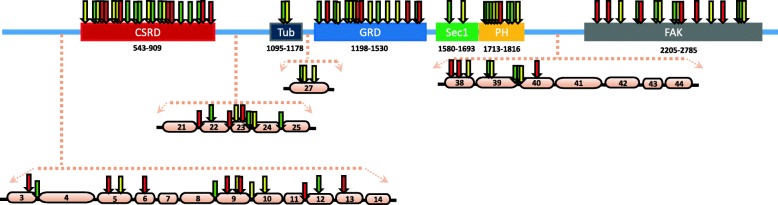
Distribution of disease-causing mutations in neurofibromin domains in patients with. Mild (Green arrow), moderate (Yellow arrow) and severe (Red arrow) phenotype. CSRD: cysteine–serine-rich domain; TBD: tubulin-binding domain; GRD: GTPase-activating protein-related domain; S1: syndecan binding domain 1; PH: pleckstrin homology domain; CTD: carboxy-terminal domain; S2: syndecan binding domain 2; SEC14/ SEC14p: Sec14-like lipid binding module. For the mutation localized outside the known domain, the specific exon localization is reported

To check whether the type of mutation influenced the observed association, we compared the prevalence of truncating and missense mutations, as well as the localization of mutations within the GRD (exons 21–27) in patients with severe phenotype and in those with moderate and mild phenotypes (Fig. 3). Comparative analysis showed no statistically significant association between the severity of phenotype and either the type of mutation or localization within the GRD (*p* > 0.05). To rule out the impact of sequence variation on PCR cynetics and probe binding, all patients were reanalyzed and the occurrence of variation located within the stretches relevant for probe/primer binding of the two TaqMan assays was excluded. Only exceptions were represented by two variants, c.4537C > T and c.7778delA, which mapped closely to the annealing site of the TaqMan assay for isoform 1 and for isoform 1/2, respectively. However, patients #13 (with mild phenotype) and #22 (with severe phenotype), despite being both heterozygous for c.4537C > T variant, showed opposite expression levels of isoform 1, under and above the average, respectively. As much as regard variant c.7778delA, case #32 (severe phenotype), who was heterozygous for this variant, showed an expression level of isoform II below the average, but the significance of results did not change after excluding this case from the analysis (*p* = 0.02). Healthy controls were not sequenced, therefore we cannot exclude the presence of rare variants occurring within the genomic stretches annealing with the TaqMan primers/probes among these subjects. However, these genomic regions do not contain common variants occurring in human populations, as reported in the Ensembl genome browser (https://www.ensembl.org/Homo_sapiens/Info/Index) or the ExAC database (http://exac.broadinstitute.org/).

## Discussion

In this study, we tested the hypothesis of a contribution of processes controlling/mediating *NF1* transcript processing to the variable phenotypic expressivity characterizing NF1 by analyzing the level of expression of the two main mRNA isoforms of the gene, which encode proteins that differ in their abilities to control Ras signaling.

NF1 is the result of loss-of-function mutations in the *NF1* gene. In this study, more than 75% of the mutations identified lead to the introduction of a premature termination codon in the coding sequence, which is in line with previous findings [12, 31]. Because of the nonsense-mediated RNA decay mechanism, many of these mutations are expected to lead to a reduction in the level of expression of the *NF1* transcript [32]. Consistently, we found that *NF1* mRNA was expressed at significantly lower levels in the peripheral blood leukocytes of NF1 patients than in healthy subjects, independently from the protein isoform that was considered and from the severity of the phenotype. Further data analysis showed that the neurofibromin isoform with higher GAP activity, isoform I, was expressed at significantly lower levels in subjects with severe phenotype respect to affected subjects with mild/moderate phenotypes, independently of the age. Moreover, when patients were compared based on the presence vs absence of LD/CD, cerebral tumors and cerebrovascular disease, analyses showed that a lower expression level of isoform I was significantly associated with occurrence of LD/CD. Such specific association is of particular relevance since isoform I is predominantly expressed in central nervous system neurons [33], and the finding that in mice, constitutional homozygous deletion of exon 23a (i.e., loss of *Nf1* mRNA isoform 1 expression in all tissues) is viable, do not affect development or cause cancer predisposition but results in spatial learning and memory defects [24, 25]. Consistent with the findings collected in mice, we did not observe any significant difference in the expression of the two *NF1* isoforms in relation to tumor formation or vascular disease. This is in line with the consideration that cell transformation is expected to require complete loss/functional inactivation of neurofibromin, which is more likely to depend upon somatic hits affecting the wild-type allele rather than events causing aberrant transcript processing. This also applies to vasculopathy and other NF1-related features, including café-au-lait spots or tibial pseudarthrosis, in which the somatic second hit has been detected in the pathologic tissue [34–36]. On the opposite, learning disability phenotypes are more thought to be related to neurofibromin haploinsufficiency and therefore could be more influenced by the balance between the expression of the two neurofibromin isoforms in the brain [6, 24, 25]. It is important to underline that the association between reduced isoform I expression and learing phenotype was still present when only subjects in pediatric age were considered. This is an extremely significant observation since the LD/CD phenotype has profound implications for the management of the disease, especially in early age, and the identification of predictive markers might be useful for the clinical management of these patients [37]. Inclusion/skipping of *NF1* exon 23a is a tightly regulated process during development, depending on the cellular context. This alternative splicing event is under complex control with many regulatory factors involved. Like other alternative exons, also *NF1* exon 23a is characterized by the presence of weak consensus sequences surrounding the exon that are not readily recognized by the splicing machinery [6]. Although not identified yet, it is possible that variation involving *cis*- and/or *trans*-acting elements controlling/participating in exon 23a retention/skipping could result in the failure of proper *NF1* transcript processing, leading to an imbalance in the distribution of the type I and type II isoforms and this in turn to phenotypic consequences in NF1 patients.

It has been widely demonstrated that dysregulation of posttranscriptional regulation, including alternative splicing, results in defective neuronal differentiation and/or synaptic connections, leading to neurodevelopmental and psychiatric disorders [38, 39]. Different genetic and chemical approaches to target components of the spliceosome to correct splicing defects have been investigated in pathological conditions including cancer and neurologic disorders. Advancements in the understanding of NF1-specific defects caused by mis-regulation of alternative splicing might increase the development of specific therapeutic options in NF1 [40–42].

## Conclusions

The present findings provide a first evidence for a role of circuits controlling *NF1* transcript processing in modulating phenotypic expressivity in NF1, and document an association between the levels of neurofibromin isoform I mRNA and the severity of phenotype and cognitive impairment. The identification of this association between specific *NF1* expression pattern and phenotype variability is remarkable and deserves further exploration. Expression studies at the protein level and in relevant tissues/cell lineages are required steps to validate the present findings.

## Supplementary information

**Additional file 1: Table S1.** List of *NF1* mutations identified in individuals with MILD NF1 phenotype.

## Data Availability

The datasets used and/or analysed during the current study are available from the corresponding author on reasonable request.

## Abbreviations

CALMs: Cafè-au-lait macules
GRD: GAP-Related domain
LD/CD: Learning disabilities/cognitive deficit
MAPK: Ras-mitogen-activated protein kinases
MPNSTs: Malignant peripheral nerve sheath tumors
NF1: Neurofibromatosis 1
NFs: Subcutaneous neurofibromas
OPGs: Optic pathway gliomas
PNFs: Plexiform neurofibromas
