# Supplementary material for: Isoform-specific NF1 mRNA levels correlate with disease severity in Neurofibromatosis type 1
Source: Orphanet J Rare Dis. 2019 Nov 15;14:261. doi: 10.1186/s13023-019-1223-1 (PMC6858644; doi:10.1186/s13023-019-1223-1)
Supplement: Supplementary file 1 — Additional file 1: Table S1. List of NF1 mutations identified in individuals with MILD NF1 phenotype. [file 13023_2019_1223_MOESM1_ESM.doc]

| Table S1. List of *NF1* mutations identified in individuals with MILD NF1 phenotype. | | |
| --- | --- | --- |
| Sample # | Nucleotide substitution^ | Amino acid change |
| 1 | c.288+1delG | p.? |
| 2 | c.889-2A>G | p.? |
| 3 | c.1863delC | p.(Cys622Valfs*9) |
| 4 | c.2014G>T | p.(Gly672*) |
| 5 | c.2041C>T | p.(Arg681*) |
| 6 | c.2125T>C | p.(Lys709Arg) |
| 7 | c.226C>T | p.(Gln756*) |
| 8 | c.2990G>C | p.(Arg997Thr) |
| 9 | c.3198-2A>G |  |
| 10 | c.3708+5G>A |  |
| 11 | c.4237A>T | p.(Arg1413*) |
| 12 | c.4267A>G | p.(Lys1423Glu) |
| 13 | c.4537C>T | p.(Arg1513*) |
| 14 | c.5242C>T | p.(Arg1748*) |
| 15 | c.5242C>T | p.(Arg1748*) |
| 16 | c.5425C>T | p.(Arg1809Cys) |
| 17 | c.5596_5596delTTTAA | p.(Asn186Lysfs*26) |
| 18 | c.5749+2T>G |  |
| 19 | c.7125delA | p.(Tyr2377Thrfs*20) |
| 20 | c.7846C>T | p.(Arg2616*) |
| 21 | c.7846C>T | p.(Arg2616*) |
| 22 | c.1318C>T | p.(Arg440*) |
| 23 | c.1887_1888delGG | p.(Val630Argfs*3) |
| 24 | c.2014G>T | p.(Gly672*) |
| 25 | c.2033dupC | p.(IIe679ASfs*21 ) |
| 26 | c.2251+1G>A^^ | p.? |
| 27 | c.3113+1G>C | p.? |
| 28 | c.3474_3475delCA | p.(Asp1158Glufs*36) |
| 29 | c.3568G>A | p.(Gly1190Ser) |
| 30 | c.4274T>C | p. (Leu1425Pro) |
| 31 | c.4829T>G | p.(Leu1610*) |
| 32 | c.5242C>T | p.(Arg1748*) |
| 33 | c.5425C>T | p.(Arg1809Cys) |
| 34 | c.5749+2T>G | p.(?) |
| 35 | c.6792C>A | p.(Tyr2264*) |
| 36 | c.6947T>G | p.(Leu2316Arg) |

^*NF1*referencesequence: NM_000267.3; ^^Patient # 26washeterozygous for an additionalpathogenicvariantin *PTPN11* c.1361C>T p.(Pro454Leu)

| Table S1. List of *NF1* mutations identified in individuals with SEVERE NF1 phenotype. | | |
| --- | --- | --- |
| Sample # | Nucleotide substitution^ | Amino acid change |
| 1 | c.277 T>C | p.(Cys93Arg) |
| 2 | c.499_502delTGTT | p.(Cys167Glnfs*10) |
| 3 | c.801G>A | p.(Trp267*) |
| 4 | c.999_1000delCA | p.(IIe334Glnfs*5) |
| 5 | c.1013A>G | p.(Asp338Gly) |
| 6 | c.1260+1604A>G | p.(?) |
| 7 | c.1470_1471 ins ATACG | p.(Tyr491Ilefs*9) |
| 8 | c.1863delC | p.(Cys622Valfs*9) |
| 9 | c.1863delC | p.(Cys622Valfs*9) |
| 10 | c.1885G>A | p.(Gly629Arg) |
| 11 | c.2041C>T | p.(Arg681*) |
| 12 | c.2041C>T | p.(Arg681*) |
| 13 | c.226C>T | p.(Gln756*) |
| 14 | c.2446C>T | p.(Arg816*) |
| 15 | c.2851-2A>G | p.(?) |
| 16 | c.2990+2T>A | p.(?) |
| 17 | c.3040A>T | p.(Lys1014*) |
| 18 | c.3721C>T | p.(Arg1241*) |
| 19 | c.4267A>G | p.(Lys1423Glu) |
| 20 | c.4267A>G | p.(Lys1423Glu) |
| 21 | c.4373delT | p.(Phe1458Serfs*4) |
| 22 | c.4537C>T | p.(Arg1513*) |
| 23 | c.5242C>T | p.(Arg1748*) |
| 24 | c.5483A>T | p.(Asp1828Val) |
| 25 | c.5543T>A | p.(Leu1848*) |
| 26 | c.5928G>A | p.(Trp1976*) |
| 27 | c.6642-3C>G | p.(?) |
| 28 | c.6709C>T | p.(Arg2273*) |
| 29 | c.6792C>A | p.(Tyr2264*) |
| 30 | c.7125delA | p.(Tyr2377Thrfs*20) |
| 31 | c.7285C>T | p.(Arg2429*) |
| 32 | c.7778delA^^^ | p.(Lys2593Argfs*10) |
| 33 | Whole gene deletion | -- |
| 34 | Whole gene deletion | -- |
| 35 | Whole gene deletion | -- |
| 36 | c.(1721+1_1722-1)_3974+1_3975-1)del | p.(?) |

^^^Patient # 8,9,32wereheterozygous for an additionalpathogenicvariant in *PTPN11* gene NM_002834.3:c.209A>G; p.(Lys70Arg) (p.8,9); NM_002834.3:c.794G>A p.(Arg265Gln) (p32).
